# Determinants of healthcare worker turnover in intensive care units: A micro-macro multilevel analysis

**DOI:** 10.1371/journal.pone.0251779

**Published:** 2021-05-14

**Authors:** Oumou Salama Daouda, Mounia N. Hocine, Laura Temime

**Affiliations:** Modélisation Epidémiologie et Surveillance des Risques Sanitaires (MESuRS) Laboratory, Conservatoire National des Arts et Métiers (Cnam), Paris, France; University of Turin, ITALY

## Abstract

**Background:**

High turnover among healthcare workers is an increasingly common phenomenon in hospitals worldwide, especially in intensive care units (ICUs). In addition to the serious financial consequences, this is a major concern for patient care (disrupted continuity of care, decreased quality and safety of care, increased rates of medication errors, …).

**Objective:**

The goal of this article was to understand how the ICU-level nurse turnover rate may be explained from multiple covariates at individual and ICU-level, using data from 526 French registered and auxiliary nurses (RANs).

**Methods:**

A cross-sectional study was conducted in ICUs of Paris-area hospitals in 2013. First, we developed a small extension of a multi-level modeling method proposed in 2007 by Croon and van Veldhoven and validated its properties using a comprehensive simulation study. Second, we applied this approach to explain RAN turnover in French ICUs.

**Results:**

Based on the simulation study, the approach we proposed allows to estimate the regression coefficients with a relative bias below 7% for group-level factors and below 12% for individual-level factors. In our data, the mean observed RAN turnover rate was 0.19 per year (SD = 0.09). Based on our results, social support from colleagues and supervisors as well as long durations of experience in the profession were negatively associated with turnover. Conversely, number of children and impossibility to skip a break due to workload were significantly associated with higher rates of turnover. At ICU-level, number of beds, presence of intermediate care beds (continuous care unit) in the ICU and staff-to-patient ratio emerged as significant predictors.

**Conclusions:**

The findings of this research may help decision makers within hospitals by highlighting major determinants of turnover among RANs. In addition, the new approach proposed here could prove useful to researchers faced with similar micro-macro data.

## Introduction

High staff turnover is currently observed in hospitals worldwide. Among nurses in particular, turnover rates have been reported to be around 20% per year, and may reach as high as 40% per year in some countries [1–3]. In a French study conducted in intensive-care units (ICUs), the yearly turnover rate was estimated at 24% among nurses and 13% among auxiliary nurses [4].

Several studies suggest that turnover is related to staff and patient outcomes. Indeed, high turnover in a hospital ward may lead to increased overtime, fatigue and stress, as well as low job satisfaction, among the remaining staff [5]. It also disrupts continuity of care, leading to decreased quality and safety of care, with potentially increased rates of medication errors, falls or other nurse-sensitive outcomes including healthcare-associated infections [5]. All in all, = this is a major concern for hospital managers, as high turnover negatively impacts hospital budgets. Indeed, this leads to substantial direct and indirect costs, and varies between courtries. Using the original Nursing Turnover Cost Calculation Methodology, yearly costs associated with turnover were estimated at $48,790 in Australia, $20,561 in the US, $26,652 in Canada and $23,711 in New Zealand [1,3]. In addition, a significant proportion of turnover costs are attributed to replacement hiring and training costs, highlighting the importance of nurse retention [1]. Hence, better understanding turnover determinants in order to implement efficient nurse retaining policies is very important.

A great deal of research has been conducted to highlight the factors contributing to the nursing shortage and voluntary turnover issues. Nurse stress and fatigue levels, as well as emotional exaustion, understaffing and poor patient safety were found to be predictors of nurses intention to leave [6–14]. In addition, several studies in different countries highlight the importance of job satisfaction and favorable work environment, with ineffective working relationships with other nurses and physicians and lack of supervisory support increasing turnover intention [7,9,11,13–18]. Burnout, work-life imbalance, moral distress, depression symptoms and workplace violence and bullying appeared to lead to higher nurse turnover intention and had an impact on nurse quality of life [12,17–25]. Moreover, social support and job satisfaction were mediators between burnout and turnover intention [21,26]. Other factors such as temporal, physical, emotional and mental workloads, salaries, night shift, rewards/recognition, job strain, job control, job complexity, advancement opportunities and the ideas of feeling valued, respected and acknowledged were also found significantly associated with turnover intention [6–8,14,16,21,24,25,27,28]. However, a better and more global understanding of how individual-level and organizational-level factors interact with turnover in hospitals is still needed, in particular in the French context where studies on hospital staff turnover are scarce [29,30].

In this context, the present work aimed at developing a predictive statistical model of the turnover rate among registered nurses and auxiliary nurses in ICUs, based on French data collected by our group [4]. The turnover rate is a quantitative outcome defined at the ICU-level, which we wished to predict using both individual-level predictors (age, number of children, stress level, etc.) and ICU-level predictors (ICU size, shiftwork organization, etc.). This requires using a multi-level modeling approach [31] specifically designed to model a group-level outcome from individual- and group-level predictors. Here, we propose an extension of the approach proposed by Croon and van Veldhoven [32] to deal with such “micro-macro” problems.

The organization of the article is as follows. First, we present the data collected in French ICUs. Second, we describe the multilevel regression model for modeling a quantitative group-level outcome (the turnover rate) using independent variables at both individual- and group- level, and validate our approach using a simulation study. Finally, we present and discuss the results obtained using the data collected in the French ICUs.

## Materials and methods

### Study data

#### Study design and population

A cross-sectional study was conducted in 30 ICUs of Paris-area hospitals of the Assistance Publique des Hôpitaux de Paris (AP-HP) between January 18, 2013 and April 2, 2013 [4]. The study population consisted of healthcare workers with patient contacts (registered nurses, auxiliary nurses, physical therapists, doctors and residents) working in the ICUs. All types of adult ICUs were included in the study (medical, surgical and polyvalent). Study data were collected using two questionnaires: a general questionnaire regarding the organizational structure of the service (completed with the nursing manager) and an individual questionnaire administered face-to-face with personnel during on-site visits to each ICU. In total, 672 healthcare workers with patient contacts (physicians, residents, registered nurses, auxiliary nurses and physical therapists) participated to the study, including 526 registered and auxiliary nurses (RANs) [4].However, here, analyses were conducted on the data obtained from RANs only as this group of healthcare workers is subject to specific organizational characteristics, not generalized across all professions.

The turnover rate, the outcome of interest, was calculated for each ICU for the year 2012 as the proportion of employees who voluntarily or involuntarily left their jobs within the ICU during that year. This outcome was computed jointly for all RANs in each ICU.

#### Ethical approval and inform consent

The study protocol was elaborated in collaboration with the AP-HP Department of medical policy and the Department of care and of paramedical activities, and was approved after presentation to the Directorate General and the Committee on hygiene, safety and working conditions. Potential participants were informed of the study by a letter of information that was sent to the head doctor and nursing manager for distribution and/or posting in the unit. Verbal consent was obtained by the interviewer at the beginning of each interview. Participants were guaranteed confidentiality and anonymity of responses. Because the study was strictly observational, did not involve medical records or biological samples, and was based on the collection of anonymous data on a voluntary basis, no further approval from an ethics committee was required by the French legislation at the time.

#### Individual-level factors

In the study questionnaire, the collected demographic characteristics of participants included age, gender, marital status, number of children, professional category and total commuting time to and from work. The questionnaire also included individual work-related characteristics such as experience duration in the profession and in the current position (years), perceived stress level and assessment of social support from supervisor and colleagues. Perceived stress was measured using the Perceived Stress Scale 10-item scale (PSS10) developed by Cohen and al. [33] and validated in French [34]. Social support was assessed using the Job Content Questionnaire, which measures the support of both colleagues and supervisor, developed and validated by Karasek et al. [35], and validated in French [36]. Further data relative to recent work history (shift assignment in the previous 30 days (day or night), frequency of schedule changes and overtime hours (both factors having four ordinal response categories, ranging from “never” (0) to “very often” (3)) and workload (average number of breaks, skipping a break in the previous three shifts due to workload (yes or no), and working quota) were also collected. Finally, fatigue was evaluated on three measures: the Nottingham Health Profile perceived health status relative to sleep (NHP-S; coded yes or no) and to energy level (NHP-E; coded yes or no), and the current fatigue state (good or bad) of the subject at the time of the interview. The NHP questionnaire was developped by Hunt et al. [37] and the French version was validated by Bucquet and Condon [38].

#### Group-level factors

ICU-level data included information on both the physical organization of the ward (number of beds, type of ICU (medical, surgical and polyvalent) and the staff-to-patient ratio computed jointly for all RANs, during days, nights, and overall) and the work organization of the staff. The latter included the type of shift work (two 12-hour shifts or three 8-hour shifts) and information on whether intermediate care beds were included within the ICU.

### Statistical modeling

#### General micro-macro model structure

To predict the turnover rate *Y*, a quantitative group-level variable, from both individual- and ICU-level factors, we used a 2-level micro-macro model. Let *G* be the total number of ICUs in which the data were collected and *n*_*g*_ the number of individuals (RANs) within the g^th^ ICU. Let *Y* be the turnover, with score *y*_*g*_ for ICU *g*.

For simplicity reasons, we first provide here the model adjusting for a single individual-level covariate (“micro” level) and a single ICU-level covariate (“macro” level). We thus want to predict *Y* by one ICU-level explanatory variable *Z* with score *z*_*g*_ for group *g* and one individual-level explanatory variable *X* with score *x*_*ig*_ for individual *i* in group *g*. In the approach proposed by Croon and van Veldhoven [32], a latent ICU-level variable *ξ* (with score *ξ*_*g*_ within ICU *g*) is associated with the individual-level variable *X*. Hence, the scores *x*_*ig*_ are viewed as indicators of the unobserved ICU scores *ξ*_*g*_. The relationship between ICU-level variables *ξ* and *Z* and the outcome *Y* may be described using a linear regression model given by the following equation:

$$y_{g}=\beta_{0}+\beta_{1}z_{g}+\beta_{2}\xi_{g}+\varepsilon_{g}$$ (1)

The error variable *ε* is assumed to be homoscedastic, that is, to have constant variance $\sigma_{\varepsilon }^{2}$ for all groups. Additionally, the relationship between the ICU-level variable *ξ* and the individual-level variable *X* may be specified by the following equation:

$$x_{ig}=\xi_{g}+\upsilon_{ig}$$ (2)

where *υ*_*ig*_ is a disturbance term, which is assumed to have a constant variance $\sigma_{\upsilon }^{2}$ for all subjects and groups and to be independent from *ε*_*g*_ and from *ξ*_*g*_.

#### Model estimation with quantitative predictors

In the case of quantitative predictors, a naive approach would be to aggregate the individual-level variable *x*_*ig*_ to the ICU level by computing its ICU-level mean $\bar{x_{g}}$ and then get a simple estimate of the parameters *β*_*j*_ based on an ordinary least square regression analysis of the regression of *y*_*g*_ on *z*_*g*_ and $\bar{x_{g}}$. However, this estimate is biased. The method proposed by Croon & van Veldhoven [32] consists in correcting this biased estimation by using an adjusted ICU mean $\tilde{x_{g}}$ instead of $\bar{x_{g}}$. This adjusted mean $\tilde{x_{g}}$ may be interpreted as the expected value of *ξ*_*g*_, taking all the observed scores on the individual- and ICU-level explanatory variables in ICU *g* into account. The exact formula for $\tilde{x_{g}}$ is available in the original article of Croon and van Veldhoven [32].

The same principle for adjusting the observed ICU means before including them in a regression model applies if several different quantitative explanatory variables at the individual and ICU levels are available, as shown in the original article of Croon and van Veldhoven [32].

#### Correction for heteroscedasticity

As ICU size was not constant, the final regression model was not homoscedastic. As in the original article of Croon and van Veldhoven [32], we corrected this by determining the heteroscedasticity-consistent covariance matrix estimator.

#### Extension to qualitative and quantitative predictors

In our case, there is a mixture in the documented covariates; some are quantitative and others qualitative. This requires an extension of the estimation method proposed by Croon and van Veldhoven for quantitative predictors only. To that aim, we transformed the qualitative variables into quantitative variables usable in the context of regression analysis, using dummy variables. For binary variables, a single dummy variable was used. For qualitative variables with *n* modalities, (*n*−1) dummy variables were defined. The original approach proposed by Croon and van Veldhoven was then applied.

#### Variable selection procedure

Variable selection was performed in two stages. In a first step, we performed a feature selection using clustering of all variables (quantitative and qualitative), separately for individual-level and ICU-level factors. The ascendant hierarchical clustering algorithm proposed in the R package ClustOfVar was used and a bootstrap approach was performed to determine the appropriate number of clusters [39]. Variables that were strongly correlated with each other were allocated to the same cluster. For each cluster, a single variable was selected based on its pertinence as a potential action lever.

In a second step, variable selection within the model was performed after computing the adjusted mean of each individual-level factor selected in the previous step. The selection was performed using regression subset selection in R applying the **regsubsets** function [40] based on Akaike’s Information Criterion (AIC) [41]. This function performs an exhaustive search of the best subsets of the variables predicting the outcome of interest.

### Simulation study

We carried out a simulation study to validate our extension of the method proposed by Croon and van Veldhoven [32] to the situation where both qualitative and quantitative predictors are used. The objective was to estimate the accuracy of the estimation of the coefficients in the adjusted regression analysis and to examine how this accuracy was affected by the number of ICUs, the ICU size, the intraclass correlation between individual-level explanatory variable and the correlation between the explanatory variables at the ICU and individual level. A detailed presentation of all simulation scenarios is presented in **S1 Appendix**. All analyses were performed using R version 3.6.0 [42].

## Results

### Simulation study

All simulation results are provided in **S1 Appendix**. The results from the adjusted regression showed lower estimation biases, irrespective of the explored simulation scenario. As expected, the benefit of using the adjusted regression rather than the unadjusted one was more important for coefficients that were associated with individual-level predictors. The approach we proposed allows to estimate the regression coefficients with a relative bias below 7% for group-level factors and below 12% for individual-level factors.

### Application to healthcare workers’ turnover

The analyzed data comprised 526 RANs, including 325 registered nurses and 201 auxiliary nurses, in a total of 30 distinct ICUs. The number of RANs per ICU differed, ranging from 7 to 32, with a mean of 17.5. The collected data on the 26 explanatory variables and the turnover is provided for both registered and auxiliary nurses in **S1** (individual-level) **and S2** (ICU-level) **Tables**. Among the 19 individual-level explanatory variables, eight of them showed a signficance difference between registered and auxiliary nurses. At group-level, staff-to-patient ratio and turnover rate revealed no significant difference between the two professions. The mean observed value of the turnover rate for RANs is equal to 0.19 (SD = 0.09).

**Fig 1:**
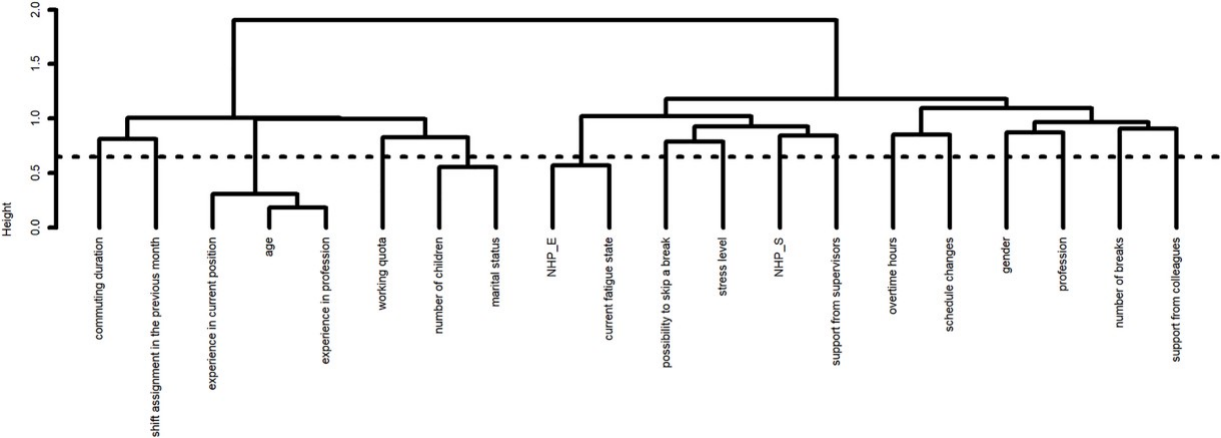
Dendrogram of the hierarchy of the 20 variables at individual-level. Dotted line depicts the best cut-off of clusters according to the bootstrap approach.

**Fig 2:**
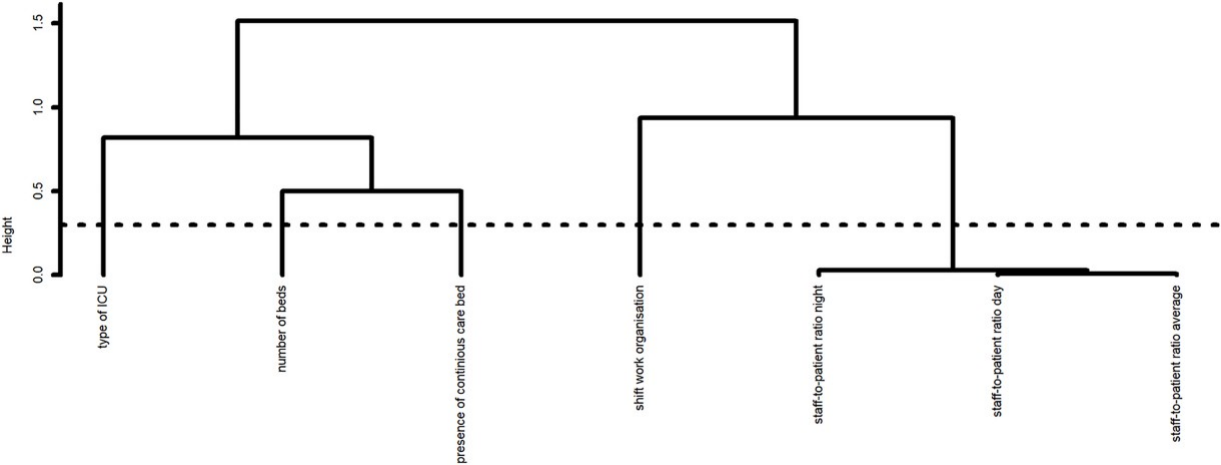
Dendrogram of the hierarchy of the 7 variables at ICU-level. Dotted line depicts the best cut-off of clusters according to the bootstrap approach.

The results of the feature selection (ascendant hierarchical clustering) first applied to reduce the dimension of the dataset are provided in **Figs 1 and 2**. The results from the bootstrap approach used to determine the stability of the partitions and therefore the number of clusters to consider are provided in **S1 and S2 Figs**. Sixteen clusters were identified for individual-level variables (**Fig 1**). The chosen variables for each cluster were: experience in profession, working quota, number of children, NHP_E, impossibility to skip a break, stress level, NHP_S, support from supervisors, overtime hours, schedules changes, commuting duration, support from colleagues, number of breaks, gender, shift assignment in the previous month and profession. Five clusters were identified for ICU-level variables (**Fig 2**); the chosen variables for each cluster were: type of ICU, number of beds, presence of intermediate care bed, shift work organization and staff-to patient ratio overall.

**Table 1** provides the results of the adjusted regression analysis to predict the turnover, based on the best model obtained after selection. This best model according to AIC included 6 individual-level variables and 4 ICU-level variables.

**Table 1:** Results of the adjusted regression analyses of turnover on individual- and ICU-level factors.

Factors	*β*	*95%* CI	*p*
**Individual-level factors (*X*)**			
Number of children	0.09	0.03; 0.15	0.005
Karasek score			
a. Support from colleagues	-0.09	-0.15; -0.02	0.005
b. Support from supervisors	-0.02	-0.04; -0.006	0.009
Duration of experience in profession (years)	-0.02	-0.03; -0.01	0.001
Impossibility to skip a break (ref = no)	0.21	0.01; 0.40	0.033
Constant schedule (ref = no)	0.11	-0.04; 0.26	0.140
**ICU-level factors (*Z*)**			
Number of beds	-0.01	-0.02; 0.005	<0.001
Presence of intermediate care bed (ref = no)	0.12	0.04; 0.19	0.002
Staff-to-patient ratio overall	0.07	0.006; 0.13	0.033
Polyvalent ICU (ref = medical)	-0.07	-0.15; -0.005	0.036

Regression coefficients (*β*) are provided with their 95% confidence interval (CI) and their p-value (*p*). *p* < 0.05 was considered statistically significant.

At the individual level, high social support from colleagues (*β* = -0.09, 95% confidence interval [CI] from -0.15 to -0.02) and supervisors (*β* = -0.02, 95%CI from -0.04 to -0.006]), as well as long durations of experience in the profession (*β* = -0.02, 95%CI from -0.03 to -0.01]), were significantly associated with lower rates of turnover. Conversely, number of children (*β* = 0.09, 95%CI from 0.03 to 0.15) and impossibility to skip a break due to workload (*β* = 0.21, 95%CI from 0.01 to 0.40])were associated with higher rates of turnover. At the ICU level, turnover was significantly associated with the number of beds (*β* = -0.01, 95%CI from -0.02 to -0.006, with lower turnover rates in large ICUs) and the type of ICU (*β* = -0.07, 95%CI from -0.15 to -0.005), lower turnover in polyvalent ICU vs. medical ICU). Higher turnover was also observed in ICUs where the staff-to-patient ratio was high (*β* = 0.07, 95%CI from 0.006 to 0.13), as well as in ICUs with intermediate care bed (*β* = 0.12, 95%CI from 0.04 to 0.19). ICCs of all individual-level factors retained in the model were significantly different from zero (see in **S3 Table**). In addition **S3 Table** provides standard error (*SE*) and the corrected standard error (*SE*_*corr*_) for all covariates. The squared multiple correlation coefficient was 0.58 means that our model explains about 58% of the variation of healthcare workers’ turnover.

## Discussion

While multilevel analysis has become increasingly popular over the last decades, most published studies have focused on modeling individual-level outcomes using individual- and group-level predictors. In this current article, we were in the opposite situation, called micro-macro, where the outcome is a group-level rather than individual level variable [32]. Micro-macro relations are present in a broad variety of researcher questions: in medical, social and behavioral sciences. Unfortunately, specific methods developed to deal with this kind of data are still very limited. Here, we proposed an extension of the Croon and van Veldhoven approach to group-level (macro) outcome, with a specific application to predict healthcare workers’ turnover. Based on the simulation study we performed, this extension allows to significantly reduce the bias in parameter estimation for micro-macro situations.

As suggested by Croon and van Veldhoven [32], micro-macro analyses “will yield stable and interpretable results only if […] the group means [of individual predictors] show sufficient variation that is not entirely due to within-group variation”. This theory was verified for each individual-level predictor selected in our model. For assessing the between ICU-level units variation for a micro variable, we fit a multilevel model with no explanatory variables but random effects for the macro unit (ICU level). We tested the effect of the variance of the random effect using the **RLRsim** package, which has a fast implementation of simulation-based tests of null hypotheses about zero variance [43,44]. For each individual-level predictor, the random effect was significant meaning that there is a variation between ICU-level units in the distribution of the individual-level predictors.

Using our approach, we identified some individual- and group-level factors determinants of RAN turnover. In particular, we found evidence that social support (from colleagues and supervisors) played a significant role on RAN turnover rate. In addition, number of children, impossibility to skip a break and long durations of experience in profession were also associated with lower RAN turnover. The latter could reflect the fact that experienced RANs are more resistant to change. All in all, our results highlight four individual and ICU-level factors less or not really present in the literature as associated to nurses turnover: the number of children, the impossibility to skip a break due to workload, the fact that the ICU is polyvalent (combining medical and surgical activities), and the presence of intermediate care beds within the ICU.

Although increasing recruitment of nurses and raising compensation may help reduce the short-term impact of turnover, researchers suggest that administrative interventions to improve the work life of RANs are more effective in the long term. Our work could provide insight into levers to improve this work life.

Moreover, Our findings are consistent with most published studies dealing with healthcare worker turnover, and in critical care in particular. Indeed, the literature highlights the importance of psychological work environment in predicting turnover [27,45,46]. Lack of social support among nurses and phycisians has been shown to be a strong predictor of turnover [7,13,16,26,47]. At the same time, low managerial support appears to favor turnover [9,14,47]. In addition, recent studies demonstrate that turnover intention is negatively correlated with years of nursing experience and years in the current position [11,48]. Job dissatisfaction or satisfaction has also frequently been reported as a determinant of intention to leave or to stay [11,13,14,21,22,27,28]. Indeed, job satisfaction of hospital nurses is closely related to work environment, structural empowerment, organizational commitment, professional commitment, job stress, patient satisfaction and patient-nurse ratios. Moreover, nurses’ health and well-being also influence experienced nurses’ decisions to leave practices [7]. In particular, even though workplace violence is associated to a higher rate of turnover intention, subjective well-being has been shown to moderate the relationship between workplace violence and nurses’ turnover, and the relationship between workplace violence and job satisfaction [26]. This moderating effect of subjective well-being has proved helpful in reducing the harm of workplace violence to nurses and in decreasing their turnover intention. Finally, it appears that nurses working conditions is associated to intention to leave, but the strength of this relationship is slightly mediated by nurse’s mental health [49].

### Methodological benefits of this work

The simulation study reported here yielded promising findings for researchers in different areas faced with micro-macro data. Micro-macro analysis is indeed currently of great interest in a wide array of fields [50,51]. Several extensions of the micro-macro method proposed by Croon and van Veldhoven had been proposed before [52–54]; for example, in 2013, Bennink and al. [53] developed the case when discrete individual-level variables are used as predictors of discrete group-level outcomes. However, none of the published extensions had treated the case where there is a mixture of quantitative and qualitative predictors whether at the group or individual levels, with a quantitative group-level outcome.

### Limitations and directions for future research

The goal of this work was double: (a) to extend the approach proposed by Croon and van Veldhoven, by allowing for qualitative individual- and group- level predictors; and (b) to apply the method to data on healthcare worker turnover. Based on our results, these goals were attained. However, it is important to acknowledge that there are some limitations in this study, whether in the developed method or in the data used.

First, the simulation study that demonstrated the performance of our methodological approach was based on the simplified situation where only 4 variables were included in the model. It could be interesting to assess the estimation biases obtained with a model including more than 4 variables with various distributions.

Second, the extension proposed here is applied to a real dataset in which RANs turnover (the group-level outcome) is explained by several group- and individual-level predictors. One of the limitations of this data is that the number of ICUs and the number of observations per ICU available for analysis were relatively small. It could be interesting to replicate the study with larger ICU-size and larger sample to improve the power for the study and to decrease coefficient biases.

Third, because of the small number of observations in the sample, we aggregated registered and auxiliary nurses in the analysis in order to achieve an adequate power for the study. However, results of separate analyses could be interesting to perform.

Fourth, our findings should be interpreted with caution when attempting to identify efficient actions to reduce turnover. Indeed, due to the cross-sectional nature of the study we used, these findings should be interpreted as reflecting statistical associations, rather than causal relationships [55]. Further longitudinal research is needed to prove relationships between turnover and these factors.

A final limitation of the study concerns the age of the data we used. Indeed, data were collected in 2013; unfortunately, we did not have access to more recent data on nurse turnover in French ICUs that would also document individual and organizational factors, as our data does. However, many of the factors we found associated with nurse turnover are consistent with the recent international literature. In addition, these factors (e.g. social support or having to skip breaks) remain pertinent to this day, actually even more so in the current context of the Covid-19 pandemic which has been shown to impact the careload, as well as the physical and mental health of healthcare workers [56]. However, the level of knowledge on turnover within hospitals would benefit from the collection of new longitudinal data.

## Conclusion

In this study, through an extension of the method initially proposed by Croon and van Veldhoven [32], we proposed an innovative methodological framework to identify the main individual- and group-level determinants of a group-level outcome. This framework could be applied to other issues within hospital wards, such as absenteeism, hand hygiene compliance or blood exposure incident rates.

In addition, our results could help hospital decision makers facing high staff turnover, by informing them on the main factors on which they may act to try and reduce turnover intention among the staff. In particular, two axes of interventions come out of our analysis: first, ensuring that hospital staff never have to skip breaks; and second, enhancing social support among the staff. The former possibly requires increasing the staff-to-patient ratio. The latter may notably be achieved by implementing solutions that enable healthcare workers to effectively and collaboratively work together and facilitate social communication and interaction.

## Supporting information

S1 Fig Cluster partition stability at individual-level. (TIFF)

S2 Fig Cluster partition stability at intensive care unit-level. (TIFF)

S1 Table Description of individual-level factors (mean ± SD for quantitative factors and n(%) for qualitative factors) for registered and auxiliary nurses. (PDF)

S2 Table Description of intensive care unit-level factors (mean ± SD for quantitative factors and n(%) for qualitative factors) for registered and auxiliary nurses. (PDF)

S3 Table Additional results of the adjusted regression analyses of turnover on individual- and intensive care unit-level factors. (PDF)

S1 Appendix Study and results of the simulation scenarios. (PDF)

S2 Appendix Dictionary of categorical variables. (PDF)

S1 File Original (French) version of the questionnaire. (PDF)

S2 File English version of the questionnaire. (PDF)

S1 Data Individual-level factors data. (CSV)

S2 Data Intensive care unit-level factors data. (CSV)
