# Supplementary figures and images for: Determinants of healthcare worker turnover in intensive care units: A micro-macro multilevel analysis
Source: PLoS One. 2021 May 14;16(5):e0251779. doi: 10.1371/journal.pone.0251779 (PMC8121288; doi:10.1371/journal.pone.0251779)

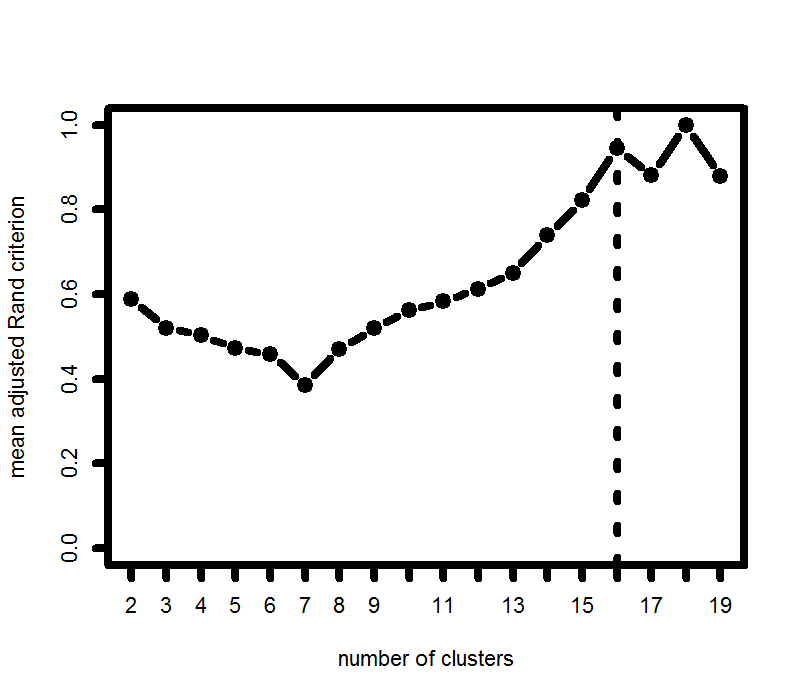

Supplement: S1 Fig — (TIFF) [file pone.0251779.s001.tiff]

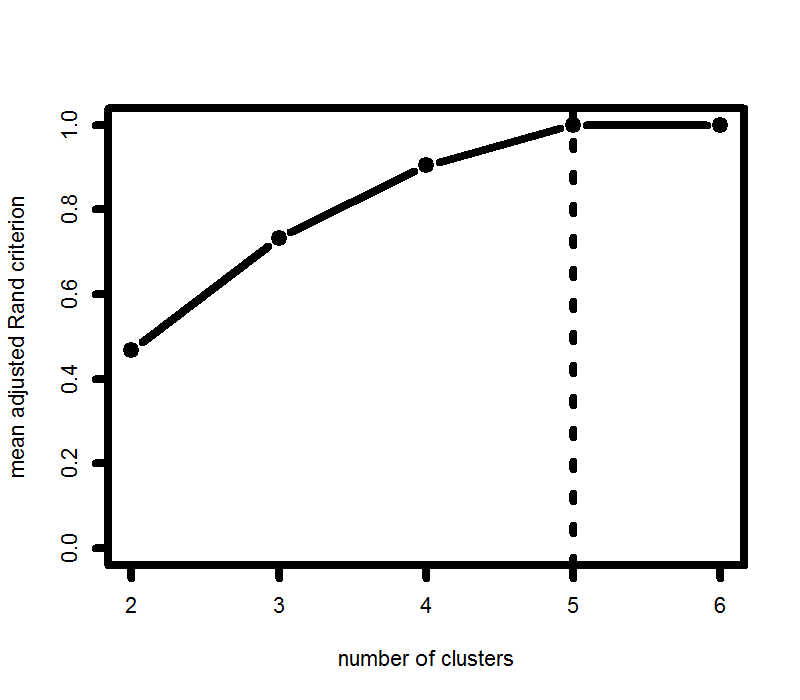

Supplement: S2 Fig — (TIFF) [file pone.0251779.s002.tiff]
