# Supplementary material for: Determinants of healthcare worker turnover in intensive care units: A micro-macro multilevel analysis
Source: PLoS One. 2021 May 14;16(5):e0251779. doi: 10.1371/journal.pone.0251779 (PMC8121288; doi:10.1371/journal.pone.0251779)
Supplement: S1 Table — (PDF) [file pone.0251779.s003.pdf]

**Individual-level factors**		**Registered nurses (N = 325)**	**Auxiliary nurses (N = 201)**	***p***
Age		30.5 ± 7.7	37.1 ± 10.4	<0.001
Experience in profession (years)		6.2 ± 6.9	10.6 ± 8.4	<0.001
Experience in current position (years)		4 ± 5.4	5.9 ± 6.7	<0.001
Working quota		98.9 ± 4.5	99.4 ± 3.3	0.11
Number of children		0.5 ± 0.9	1.1 ± 1.2	<0.001
Stress level		23 ± 4.5	21.9 ± 5	0.43
Support from supervisors		6.7 ± 2.5	6.9 ± 2.3	0.46
Support from colleagues		9.7 ± 1.9	9.3 ± 2	0.01
Commuting duration (minutes)		63.8 ± 39.2	81.6 ± 52.9	<0.001
Number of breaks		2 ± 1.1	1.9 ± 1	0.17
Gender	Male	81 (24.9)	57 (28.4)	0.44
	Female	244 (75.1)	144 (71.6)	
Marital status	Single	134 (41.2)	72 (35.8)	<0.001
	Married or in a couple	187 (57.5)	111 (55.2)	
	Separated or divorced	4 (1.2)	18 (9)	
NHP-E (energy difficulties)	Yes	127 (39.1)	97 (48.3)	0.04
	No	198 (60.3)	104 (51.7)	
NHP-S (sleep difficulties)	Yes	196 (60.3)	133 (66.2)	0.20

Individual-level factors		Registered nurses (N = 325)	Auxiliary nurses (N = 201)	*p*
	No	129 (39.7)	68 (33.8)	
Current fatigue state	Good	176 (54.1)	97 (48.3)	0.22
	Bad	149 (45.9)	104 (51.7)	
Overtime hours	Never	145 (44.6)	109 (54.2)	
	Occasionally	140 (43.1)	85 (42.3)	0.003
	Often	37 (11.4)	7 (3.5)	
	Very often	3 (0.9)	0 (0)	
Schedules changes	Never	233 (71.7)	135 (67.2)	0.62
	Occasionally	79 (24.3)	57 (28.4)	
	Often	10 (3.1)	8 (4)	
	Very often	3 (0.9)	1 (0.5)	
Shift assignment in the previous month	Day	174 (53.5)	110 (54.7)	0.86
	Night	151 (46.5)	81 (45.3)	
Impossibility to skip a break	Yes	179 (55.1)	127 (63.2)	0.08
	No	146 (44.9)	74 (36.8)	

Two-sided t.test were performed for quantitative variables and Chi^2 tests were performed for qualitative variables
