# Supplementary material for: Determinants of healthcare worker turnover in intensive care units: A micro-macro multilevel analysis
Source: PLoS One. 2021 May 14;16(5):e0251779. doi: 10.1371/journal.pone.0251779 (PMC8121288; doi:10.1371/journal.pone.0251779)
Supplement: S2 Table — (PDF) [file pone.0251779.s004.pdf]

**ICU-level factors**		**Registered nurses (N =325)**	**Auxiliary nurses (N =201)**	***p***
**Turnover**		**0.24 ± 0.11**	**0.13 ± 0.1**	<0.001
Staff-to-patient ratio – day		0.38 ± 0.05	0.25 ± 0.06	<0.001
Staff-to-patient ratio - night		0.36 ± 0.05	0.21 ± 0.04	<0.001
Staff-to-patient ratio – overall		0.37 ± 0.05	0.23 ± 0.05	<0.001
Number of beds		15.9 ± 6.5		
Type of ICU	Medical	11 (36.7)		
	Surgical	12 (40.0)		
	Polyvalent (medical and surgical)	7 (23.3)		
Presence of continuous care beds	Yes	14 (46.7)		
	No	16 (53.3)		
Shift work organization	Two 12-hour shifts	25 (83.3)		
	Three 8-hour shifts	5 (16.7)		

Two-sided t.test were performed for quantitative variables and Chi² tests were performed for qualitative variables
