# Supplementary material for: Determinants of healthcare worker turnover in intensive care units: A micro-macro multilevel analysis
Source: PLoS One. 2021 May 14;16(5):e0251779. doi: 10.1371/journal.pone.0251779 (PMC8121288; doi:10.1371/journal.pone.0251779)
Supplement: S3 Table — (PDF) [file pone.0251779.s005.pdf]

Factors	β	SE	SE_{corr}	ICC (95% CI)
**Individual-level factors (X)**				
Number of children	0.09	0.03	0.03	0.03 [0; 0.07]
Karasek score				
a. Support from colleagues	-0.09	0.03	0.02	0.07 [0.02; 0.14]
b. Support from supervisors	-0.02	0.008	0.007	0.23 [0.12; 0.34]
Duration of experience in profession (years)	-0.02	0.006	0.006	0.09 [0.04; 0.17]
Impossibility to skip a break (ref = no)	0.21	0.09	0.10	0.12 [0.04; 0.20]
Constant schedule (ref = no)	0.11	0.07	0.06	0.10 [0.03; 0.19]
**ICU-level factors (Z)**				
Number of beds	-0.01	0.002	0.003	
Presence of intermediate care bed (ref = no)	0.12	0.03	0.03	
Staff-to-patient ratio overall	0.07	0.03	0.03	
Polyvalent ICU (ref = medical)	-0.07	0.04	0.04	

β, the regression coefficients; SE, standard error; SE_{corr}, the adjusted standard error after application of the Croon and van Veldhoven correction; and ICC (95% CI) the intraclass correlation for the individual-level explanatory variables and their 95% confidence interval.
