# Supplementary material for: Determinants of healthcare worker turnover in intensive care units: A micro-macro multilevel analysis
Source: PLoS One. 2021 May 14;16(5):e0251779. doi: 10.1371/journal.pone.0251779 (PMC8121288; doi:10.1371/journal.pone.0251779)
Supplement: S1 Appendix — (PDF) [file pone.0251779.s006.pdf]

Simulation study

We carried out a simulation study to validate our extension of the method proposed by Croon and van Veldhoven [30] to the situation where both qualitative and quantitative predictors are used. The objective was to estimate the accuracy of the estimation of the coefficients in the adjusted regression analysis and to examine how this accuracy was affected by the number of ICUs, the ICU size, the intraclass correlation between individual-level explanatory variable and the correlation between the explanatory variables at the ICU and individual level. To that aim, we generated data using two ICU-level explanatory variables (Z_1, Z_2) and two individual-level explanatory variables $(\xi_1,\ \xi_2)$. Simulation scenarios were based on the following regression equation:

$$y_g = \beta_0 + \beta_1 z_{1g} + \beta_2 z_{2g} + \beta_3 \xi_{1g} + \beta_4 \xi_{2g} + \varepsilon_g \qquad (3)$$

We assumed that:

- $\beta_0 = \beta_1 = \beta_2 = \beta_3 = \beta_4 = 0.3$;
- Z_1 and ξ_1 were both quantitative covariates normally distributed with mean zero and variance $\sigma^2_{\xi_1} = \sigma^2_{z_1} = 1$;
- the correlation between Z_1 and ξ_1 could be at two levels: $\rho_{z_1\xi_1} = 0$ and $\rho_{z_1\xi_1} = 0.3$;
- Z_2 was a binary covariate following a Bernoulli distribution with probability 0.2;
- ε_g (the residual term for ICU g) was normally distributed with mean zero and variance $\sigma^2_\varepsilon = 0.2$;

The individual score of the quantitative variable x_{i1g} was obtained using $x_{i1g} = \xi_{1g} + \upsilon_{ig}$. υ_{ig} was assumed normally distributed with mean zero and variance varying between 4 and 9, as proposed by Croon and van Veldhoven [30]. Since the intraclass correlation coefficient

(ICC) for a quantitative variable is defined as $\rho_{X_1} = \frac{\sigma^2_{\xi_1}}{\sigma^2_{\xi_1} + \sigma^2_{v}}$, this meant that ρ_{X_1} was equal to either 0.1 or 0.2. On the other hand, the individual score for the binary variable x_{i2g} was obtained using $x_{i2g} \sim$ Bernoulli(ξ_{2g}) where logit (ξ_{2g}) was assumed to follow a normal distribution. Thus, logit$\left(\xi_{2g}\right) = ln\left(\frac{\xi_{2g}}{1-\xi_{2g}}\right) = u_g$, where $u_g \sim \mathcal{N}(0, \sigma^2_u)$. In this case, the ICC is computed as $\rho_{X_2} = \frac{\sigma^2_u}{\sigma^2_u + \pi/3}$ [37,38]. We varied σ^2_u so that ρ_{x2} was equal to ρ_{X_1}(that is, either 0.1 or 0.2). In addition, we explored two levels for the total number of ICUs: either $G = 30$ or $G = 60$. Three options were also assessed regarding the number of observations in each ICU. The first two options assumed a constant ICU size, at either $n_g = 20$ or $n_g = 40$. In the third option, ICU size was varied by randomly allocating either 20 or 40 observations to each ICU with equal probability. For each set of parameters, 1000 data samples where generated. For each sample, both an unadjusted and an adjusted regression analysis were carried out. The outcomes of interest in this simulation study were the relative bias in the estimation of the five regression coefficients and the coverage rate of the true value (0.3) of each coefficient β_i by the estimated confidence interval. All analyses were performed using R version 3.6.0 [39].

Results

The table 1 provides the results of the simulation study including the estimation biases for all model parameters obtained using both standard (unadjusted) regression and the extended adjusted regression approach we propose**.**

No.	$\rho_{Z_1\xi_1}$	ρ_{X_1}= ρ_{X_2}	G	n_g	β_0		β_1		β_2		β_3		β_4	
					**UR**	**AR**	**UR**	**AR**	**UR**	**AR**	**UR**	**AR**	**UR**	**AR**
1	0.0	0.1	30	20	14.7	4.5	0.0	2.5	-0.4	7.3	29.5	11.7	-32.4	-11.4
2	0.0	0.1	30	40	10.4	2.1	-1.1	-1.0	-1.7	-1.2	-17.4	5.0	-31.8	-3.4
3	0.0	0.1	30	Mix	18.0	1.0	0.9	1.3	-5.9	-5.7	-22.5	9.0	-31.0	-3.5
4	0.0	0.1	60	20	20.3	-0.8	0.0	0.0	2.0	2.3	-29.9	6.5	-44.2	-2.2
5	0.0	0.1	60	40	10.4	-1.3	-0.1	-0.2	-0.6	-1.1	-17.3	2.8	-23.6	0.1
6	0.0	0.1	60	Mix	16.9	-1.2	0.0	-0.0	-1.8	-1.4	-24.3	2.5	-33.6	2.2
7	0.0	0.2	30	20	10.1	-4.6	-0.1	-0.1	1.5	1.1	-15.3	4.7	-22.7	7.2
8	0.0	0.2	30	40	6.9	-0.5	2.4	-0.9	1.4	1.6	-8.0	3.4	-12.2	2.6
9	0.0	0.2	30	Mix	8.7	-2.2	0.0	-0.1	-0.0	0.2	-12.5	3.7	-23.6	-4.5
10	0.0	0.2	60	20	12.8	0.7	1.5	1.5	-2.0	-1.7	-16.5	1.5	-24.2	-0.6
11	0.0	0.2	60	40	7.6	0.7	-0.7	-0.8	-0.6	-0.6	-8.3	1.4	-12.4	1.4
12	0.0	0.2	60	Mix	9.3	0.1	0.7	0.8	-0.4	-0.8	-11.1	2.7	-17.5	1.1
13	0.3	0.1	30	20	18.3	-3.1	10.0	-4.7	-0.8	-2.7	-32.1	10.1	-40.5	8.5
14	0.3	0.1	30	40	18.7	-0.8	8.7	-1.5	-0.1	0.5	-22.7	6.4	-34.9	3.2
15	0.3	0.1	30	Mix	14.6	-4.4	7.9	-2.8	3.1	2.7	-24.8	9.0	-30.2	5.3
16	0.3	0.1	60	20	19.0	-2.7	10.1	-1.5	3.1	4.4	-32.3	7.0	-41.2	1.3
17	0.3	0.1	60	40	11.6	-0.0	5.5	-1.0	0.7	0.4	-19.6	2.1	-25.4	-2.1
18	0.3	0.1	60	Mix	15.1	-2.0	6.1	-2.8	-1.4	-2.1	-26.0	3.8	-28.0	7.4
19	0.3	0.2	30	20	8.9	-4.5	2.9	-3.7	-1.2	-1.3	-16.8	5.2	-22.9	4.4
20	0.3	0.2	30	40	6.0	-2.0	4.0	0.7	1.7	2.0	-10.4	0.7	-14.7	1.0
21	0.3	0.2	30	Mix	10.5	-1.0	3.7	-1.3	-3.0	-2.7	-10.8	6.1	-18.2	4.6
22	0.3	0.2	60	20	10.2	-3.0	4.8	-1.1	-1.9	-1.6	-17.4	2.5	-19.4	6.8
23	0.3	0.2	60	40	5.7	-0.1	3.5	0.3	0.7	0.6	-10.0	0.4	-14.0	-0.6
24	0.3	0.2	60	Mix	7.2	-2.5	5.8	1.4	0.6	0.7	-13.4	1.7	14.2	5.1
**Bias averaged over all conditions**					**12.2**	**-1.2**	**3.2**	**-0.6**	**-0.3**	**0.04**	**-16.2**	**4.6**	**-24.4**	**1.4**

$\rho_{Z_1\xi_1}$, correlation of explanatory ICU-level variable Z_1 and individual-level explanatory variable ξ_1; $\rho_{X_1}$and ρ_{X_2}, intraclass correlation of X_1 and X_2; G , number of ICUs; n_g, ICU size.

Table 1. Mean percentages of bias for the five coefficients from the unadjusted (UR) and adjusted (AR) regression analyses, based on 1000 simulated datasets.

No.	$\rho_{Z_1\xi_1}$	$\rho_{X_1}=\rho_{X_2}$	G	n_g	β_0		β_1		β_2		β_3		β_4	
					**UR**	**AR**	**UR**	**AR**	**UR**	**AR**	**UR**	**AR**	**UR**	**AR**
1	0.0	0.1	30	20	94.9	94.8	94.5	93.6	95.0	93.7	78.7	92.1	95.2	93.9
2	0.0	0.1	30	40	94.8	93.4	95.6	95.2	95.2	94.8	92.0	93.9	95.1	93.7
3	0.0	0.1	30	Mix	95.4	95.0	94.8	94.3	94.7	94.0	84.1	93.0	95.1	94.0
4	0.0	0.1	60	20	95.7	93.9	96.0	95.1	95.7	95.1	60.4	92.2	94.4	94.6
5	0.0	0.1	60	40	94.8	95.3	96.0	96.1	94.5	94.4	85.3	93.3	94.8	95.1
6	0.0	0.1	60	Mix	93.8	94.5	94.6	93.7	95.9	95.3	75.4	93.0	94.2	94.5
7	0.0	0.2	30	20	94.5	94.6	94.7	94.2	94.6	94.1	92.8	95.0	94.4	94.5
8	0.0	0.2	30	40	94.4	94.6	95.1	95.1	95.1	94.9	93.4	94.8	94.6	94.6
9	0.0	0.2	30	Mix	95.2	94.6	95.3	95.1	95.7	95.2	91.4	95.0	94.6	94.7
10	0.0	0.2	60	20	94.9	95.2	95.0	94.4	94.2	94.8	87.5	94.5	94.0	94.7
11	0.0	0.2	60	40	95.6	95.3	96.2	96.0	94.1	93.6	93.2	95.5	95.4	95.8
12	0.0	0.2	60	Mix	94.9	95.0	94.4	94.2	95.3	95.6	90.9	94.6	95.7	95.3
13	0.3	0.1	30	20	93.3	93.3	94.7	95.0	96.0	94.9	79.1	93.3	94.1	93.7
14	0.3	0.1	30	40	95.7	95.3	94.7	94.5	94.2	94.0	90.3	95.5	95.6	95.3
15	0.3	0.1	30	Mix	95.2	94.5	95.0	93.7	95.1	94.2	83.7	93.7	94.1	93.7
16	0.3	0.1	60	20	93.7	93.2	92.7	93.8	94.2	93.2	59.2	94.2	93.4	93.8
17	0.3	0.1	60	40	94.3	94.1	94.6	95.9	95.2	94.5	81.5	94.8	94.2	94.5
18	0.3	0.1	60	Mix	95.7	96.0	94.4	94.8	95.5	95.0	72.9	93.3	95.5	95.6
19	0.3	0.2	30	20	94.5	94.8	94.2	93.3	95.3	95.0	91.4	93.9	94.9	95.1
20	0.3	0.2	30	40	95.5	95.6	96.3	96.1	95.2	94.7	95.0	96.2	95.8	95.4
21	0.3	0.2	30	Mix	94.9	94.5	94.1	93.7	93.5	94.0	93.2	95.7	94.4	94.3
22	0.3	0.2	60	20	94.7	95.1	94.4	94.8	94.7	95.4	84.6	94.5	95.1	95.6
23	0.3	0.2	60	40	95.9	96.1	95.5	95.0	94.7	95.0	93.9	95.2	96.3	96.3
24	0.3	0.2	60	Mix	95.9	96.0	94.9	95.1	95.6	94.8	91.8	94.1	95.3	95.7

$\rho_{Z_1\xi_1}$, correlation of explanatory ICU-level variable Z_1 and individual-level explanatory variable ξ_1; $\rho_{X_1}$and ρ_{X_2}, intraclass correlation of X_1 and X_2; G , number of ICUs; n_g, ICU size.

Table 2. Coverage rate of the true value of each coefficient by the estimated confidence interval from the unadjusted (UR) and adjusted (AR) regression analyses, based on 1000 simulated dataset

The results from the adjusted regression showed lower estimation biases, irrespective of the explored simulation scenario. As expected, the benefit of using the adjusted regression rather than the unadjusted one was more important for coefficients β_3 and β_4, that were associated with individual-level predictors. In addition, the number of observations per ICU n_g had a significant impact on all estimated biases, with lower biases for large n_g values. The bias in the estimation of β_1 was also lower for high values of the correlation $\rho_{Z_1\xi_1}$; and the biases in the estimation of β_3 and β_4 were smaller for high values of the intra-class correlation ρ_{X_1}. No other explored parameter had a significant impact on the estimated biases. Overall, estimation biases remained below 5% for β_0 and β_1, below 7% for β_2, and below 12% for β_3 and β_4, suggesting that the adjusted regression approach was acceptable. In addition, **Table 2** provides the coverage rate of the true value (0.3) of each coefficient β_i by the estimated confidence interval. It appears that, in the adjusted regression, this percentage is always over 92% irrespective of the explored simulation scenario.
