# Supplementary material for: Determinants of healthcare worker turnover in intensive care units: A micro-macro multilevel analysis
Source: PLoS One. 2021 May 14;16(5):e0251779. doi: 10.1371/journal.pone.0251779 (PMC8121288; doi:10.1371/journal.pone.0251779)
Supplement: S2 Appendix — (PDF) [file pone.0251779.s007.pdf]

Dictionary for categorical variables

a) Individual-level factors

Possibility to skip a break: 0 (yes) and 1(no)

NHP_S (presence of sleep difficulties): 0 (no) and 1 (yes)

NHP_E (presence of energy difficulties): 0 (no) and 1 (yes)

Current fatigue state: 0 (good) and 1 (bad)

Marital status: single (0), married or in couple (1) and separated or divorced (2)

Schedule changes and overtime hours: 0 (never), 1 (occasionally), 2 (often), 3 (very often)

Shift assignment in the previous month: 0 (night) and 1 (day)

Profession: 0 (auxiliary nurses) and 1 (registered nurses)

b) Intensive care unit-level factors

Shift work organization: 0 (2*12h) and 1 (3*8h)

Type of ICU: 0 (medical), 1 (surgical) and 2 (polyvalent = medical + surgical)

Presence of continuous care bed: 0 (no) and (yes)
