# Supplementary material for: Determinants of healthcare worker turnover in intensive care units: A micro-macro multilevel analysis
Source: PLoS One. 2021 May 14;16(5):e0251779. doi: 10.1371/journal.pone.0251779 (PMC8121288; doi:10.1371/journal.pone.0251779)
Supplement: S1 File — (PDF) [file pone.0251779.s008.pdf]

QUESTIONNAIRE RELATIF AU SERVICE

1. Hôpital et type de service de réanimation adulte

Hôpital Cliquez ou appuyez ici pour entrer du texte.
Site (à remplir par le Cnam) Cliquez ou appuyez ici pour entrer du texte.

Type d'activité de réanimation

Médicale ☐ Chirurgicale ☐ Polyvalente ☐

2. Lits, chambres et salles

No. total de lits de réanimation ☐ d'USC ☐ No. de salles ☐

Types et nombre de chambre

☐ Individuel Nombre ☐
☐ Double Nombre ☐
☐ Box Nombre de boxs ☐ Nombre de lits box 1 ☐ box 2 ☐ box 3 ☐

Salle de repos à disposition ?

☐ Oui ☐ Non

3a. Organisation des horaires

Infirmiers: ☐ 2-12 ☐ 3-8

Aides-soignants: ☐ 2-12 ☐ 3-8

Affectation jour ou nuit: ☐ Fixe ☐ Variable

Heure de prise de poste

☐ Matin ☐ Garde (si 3-8) ☐ Nuit

3b. Organisation et déplacements

Avec quelle fréquence votre personnel est amené à sortir du service (examens, brancardages, …)

☐ Jamais ☐ Occasionnellement ☐ Souvent ☐ Très souvent

4. Nombre de soignants affectés au service (en équivalent Temps Plein)

Total affecté au service

Médecins	Cadres	Infirmiers	Aides-soignants	Kinésithérapeute
☐	☐	☐	☐	☐

Affectés le jour

Médecins	Cadres	Infirmiers	Aides-soignants	Kinésithérapeute
☐	☐	☐	☐	☐

Affectés de garde (3-8)

Médecins ☐ Cadres ☐ Infirmiers ☐ Aides-soignants ☐ Kinésithérapeute ☐

Affectés la nuit

Médecins ☐ Cadres ☐ Infirmiers ☐ Aides-soignants ☐ Kinésithérapeute ☐

5. <u>Pour pouvoir estimer le turnover des soignants, le nombre total de départs sur l'année</u>

Infirmiers ☐ Aides-soignants ☐

6a. <u>Du fait d'absences non prévues, avec quelle fréquence avez-vous recours à des personnes extérieures au service (au sein de l'hôpital) ?</u>

Infirmiers

☐ Jamais ☐ Occasionnellement ☐ Souvent ☐ Très souvent

Aides-soignants

☐ Jamais ☐ Occasionnellement ☐ Souvent ☐ Très souvent

6b. <u>Du fait d'absences non prévues, avec quelle fréquence avez-vous recours à l'intérim ?</u>

Infirmiers

☐ Jamais ☐ Occasionnellement ☐ Souvent ☐ Très souvent

Aides-soignants

☐ Jamais ☐ Occasionnellement ☐ Souvent ☐ Très souvent

<u>QUESTIONNAIRE RELATIF AU PERSONNEL SOIGNANT</u>

1. <u>Hôpital et service</u>

Hôpital Cliquez ou appuyez ici pour entrer du texte.
Site (à remplir par le Cnam) Cliquez ou appuyez ici pour entrer du texte.

<u>Type d'activité de réanimation</u>

Médicale ☐ Chirurgicale ☐ Polyvalente ☐

2. <u>Date et heure de remplissage du questionnaire</u>

Date/Heure ☐ Ordre de passage ☐ Code Questionnaire ☐

3a. <u>Profession</u>

☐Médecin (senior) ☐Médecin (interne) ☐Infirmier ☐Aide-soignant ☐Kinésithérapeute

3b. Etes-vous affecté (e) à

☐Réanimation ☐USC ☐les 2

4. Sexe

☐Homme ☐Femme

5. Age et ancienneté

Age (années révolues) ☐

Ancienneté dans le métier ☐

Ancienneté au poste ☐

6. Quotité travaillée

En % TP ☐

7a. Au cours des 30 derniers jours, quelle était votre horaire d'affectation principal ?

☐Jour ☐Garde (si 3-8) ☐Nuit

7b. Au cours des 30 derniers jours, étiez-vous affecté à cet horaire

☐Tout le temps ☐Majoritairement

8. Historique de travail

Heure de prise de poste ce jour/nuit ☐

Etiez-vous au travail lors des 3 derniers jours ?

J-3	J-2	J-1
☐Oui ☐Non	☐Oui ☐Non	☐Oui ☐Non

9. Pauses prises habituellement au cours d'une période de travail

Nombre de pauses ☐ Durée moyenne (min) ☐

10. Au cours des 3 derniers jours travaillés, avez-vous renoncé à une pause suite à une surcharge de travail ?

☐Oui ☐Non

11. Statut marital

☐Célibataire ☐Marié ou en couple ☐Séparé-veuf-divorcé

12. Avez-vous des enfants ?

☐Oui ☐Non

Si oui, nombre d'enfants : Moins de 2 ans ☐
De 2 à 10 ans ☐
Plus de 10 ans ☐

13. Temps de transport quotidien

Temps total (min) ☐

14. **<u>Nottingham Health Profile</u> : Pour chacune des affirmations suivantes indiquez si elles décrivent votre état actuel (proche sur la vie en général)**
Je me sens tout le temps fatigué (e)
☐Oui ☐Non

Je prends des médicaments pour dormir
☐Oui ☐Non

Tout me demande un effort
☐Oui ☐Non

Je me réveille très tôt et j'ai du mal à dormir
☐Oui ☐Non

Je reste éveillé (e) une grande partie de ma période de sommeil
☐Oui ☐Non

Je me fatigue vite
☐Oui ☐Non

Je mets beaucoup de temps à m'endormir
☐Oui ☐Non

Je dors mal
☐Oui ☐Non

15. **<u>Comment sentez-vous en ce moment ?</u>**
☐Epuisé (e) ☐Fatigué (e) ☐Plutôt en forme ☐En excellente forme

16. **<u>Vous êtes-vous réveillé aujourd'hui avec une sensation de fatigue ?</u>**
☐Oui ☐Non

17. **<u>Historique de sommeil au cours des derniers 24h</u>**
Heure de réveil ce jour (hors sieste) ☐
Heure de coucher ☐
Durée totale des siestes (h :mn) ☐

18. **<u>Avec quelle fréquence vous arrive-t-il de …</u>**
Travailler à des horaires différents de ceux initialement prévus
☐Jamais ☐Occasionnellement ☐Souvent ☐Très souvent

Effectuer des heures supplémentaires
☐Jamais ☐Occasionnellement ☐Souvent ☐Très souvent

19. **<u>PSS-10</u> : Au cours du dernier mois, combien de fois …**
Avez-vous été dérangé (e) par un évènement inattendu
☐Jamais ☐Presque jamais ☐Parfois ☐Assez-souvent ☐Souvent

Vous a-t-il semblé difficile de contrôler les choses importantes de votre vie ?
☐Jamais ☐Presque jamais ☐Parfois ☐Assez-souvent ☐Souvent

Vous êtes-vous senti(e) nerveux(se) ou stressé(e) ?

☐Jamais ☐Presque jamais ☐Parfois ☐Assez-souvent ☐Souvent

Vous êtes-vous senti(e) confiant(e) à prendre en main vos problèmes personnels ?
☐Jamais ☐Presque jamais ☐Parfois ☐Assez-souvent ☐Souvent

Avez-vous senti que les choses allaient comme vous le vouliez ?
☐Jamais ☐Presque jamais ☐Parfois ☐Assez-souvent ☐Souvent

Avez-vous pensé que vous ne pouviez pas assumer toutes les choses que vous deviez faire ?
☐Jamais ☐Presque jamais ☐Parfois ☐Assez-souvent ☐Souvent

Avez-vous été capable de maîtriser votre énervement ?
☐Jamais ☐Presque jamais ☐Parfois ☐Assez-souvent ☐Souvent

Avez-vous senti que vous dominiez la situation ?
☐Jamais ☐Presque jamais ☐Parfois ☐Assez-souvent ☐Souvent

Vous êtes-vous senti(e) irrité(e) parce que évènements échappaient à vôtre contrôle ?
☐Jamais ☐Presque jamais ☐Parfois ☐Assez-souvent ☐Souvent

Avez-vous trouvé que les difficultés s'accumulaient à un tel point que vous ne pouviez les contrôler ?
☐Jamais ☐Presque jamais ☐Parfois ☐Assez-souvent ☐Souvent

20. Questionnaire de Karasek : votre opinion sur votre situation de travail
Mon supérieur se sent concerné par le bien-être de ses subordonnés
☐Pas du tout d'accord ☐Pas d'accord ☐D'accord ☐Tout à fait d'accord

Mon supérieur prête attention à ce que je dis
☐Pas du tout d'accord ☐Pas d'accord ☐D'accord ☐Tout à fait d'accord

Mon supérieur m'aide à mener ma tâche à bien
☐Pas du tout d'accord ☐Pas d'accord ☐D'accord ☐Tout à fait d'accord

Mon supérieur réussit facilement à faire collaborer ses subordonnés
☐Pas du tout d'accord ☐Pas d'accord ☐D'accord ☐Tout à fait d'accord

Les collègues avec qui je travaille sont des gens professionnellement compétents
☐Pas du tout d'accord ☐Pas d'accord ☐D'accord ☐Tout à fait d'accord

Les collègues avec qui je travaille me manifestent de l'intérêt
☐Pas du tout d'accord ☐Pas d'accord ☐D'accord ☐Tout à fait d'accord

Les collègues avec qui je travaille sont amicaux
☐Pas du tout d'accord ☐Pas d'accord ☐D'accord ☐Tout à fait d'accord

Les collègues avec qui je travaille m'aident à mener les tâches à bien
☐Pas du tout d'accord ☐Pas d'accord ☐D'accord ☐Tout à fait d'accord
