# Supplementary material for: Determinants of healthcare worker turnover in intensive care units: A micro-macro multilevel analysis
Source: PLoS One. 2021 May 14;16(5):e0251779. doi: 10.1371/journal.pone.0251779 (PMC8121288; doi:10.1371/journal.pone.0251779)
Supplement: S2 File — (PDF) [file pone.0251779.s009.pdf]

# ICU-LEVEL QUESTIONNAIRE

## 1. Hospital and type of adult intensive care unit

Hospital Cliquez ou appuyez ici pour entrer du texte.
Location Cliquez ou appuyez ici pour entrer du texte.

**Type of intensive care unit activity**

Medical ☐ Surgical ☐ Polyvalent ☐

## 2. Beds, rooms and wards

No. of ICU beds ☐ No. of continuous care unit beds ☐ No. of rooms ☐

**Types and number of rooms**

☐ Single Number ☐
☐ Double Number ☐
☐ Cubicles Number of cubicles ☐ Number of beds in cubicle1 ☐ cubicle 2 ☐
cubicle 3 ☐

**Break room available?**

☐ Yes ☐ No

## 3a. Shift work organization

| Nurses | Auxiliary nurses | Day or night assignment |
|---|---|---|
| ☐ 2-12 ☐ 3-8 | ☐ 2-12 ☐ 3-8 | ☐ Fixed ☐ Variable |

**Start time of the shift**

☐ Morning ☐ Afternoon (if 3-8) ☐ Night

## 3b. Organization and movements

**How often does your staff have to go out of the unit (e.g. for examinations, stretcher transportation? ...)**

☐ Never ☐ Occasionally ☐ Often ☐ Very often

## 4. Number of healthcare workers allocated to the unit (in full-time equivalent)

**Total allocated to the unit**

| Physicians | Nurse managers | Nurses | Auxiliary nurses | Physical Therapist |
|---|---|---|---|---|
| ☐ | ☐ | ☐ | ☐ | ☐ |

**Allocated during daytime**

| Physicians | Nurse managers | Nurses | Auxiliary nurses | Physical Therapist |
|---|---|---|---|---|
| ☐ | ☐ | ☐ | ☐ | ☐ |

**Allocated during afternoons (3-8)**

| Physicians | Nurse managers | Nurses | Auxiliary nurses | Physical Therapist |
|---|---|---|---|---|
| ☐ | ☐ | ☐ | ☐ | ☐ |

**Allocated during nighttime**

| Physicians | Nurse managers | Nurses | Auxiliary nurses | Physical Therapist |
|---|---|---|---|---|
| ☐ | ☐ | ☐ | ☐ | ☐ |

5. **In order to estimate the turnover of healthcare workers, the total number of departures over the year**

Nurses ☐ Auxiliary nurses ☐

6a. **Due to unplanned absences, how often do you use staff from outside the unit (but from within the hospital)?**

**Nurses**

☐ Never ☐ Occasionally ☐ Often ☐ Very often

**Auxiliary nurses**

☐ Never ☐ Occasionally ☐ Often ☐ Very often

6b. **Due to unplanned absences, how often do you use temporary staff?**

**Nurses**

☐ Never ☐ Occasionally ☐ Often ☐ Very often

**Auxiliary nurses**

☐ Never ☐ Occasionally ☐ Often ☐ Very often

# HEALTHCARE WORKER QUESTIONNAIRE

1. **Hospital and unit**
Hospital Cliquez ou appuyez ici pour entrer du texte.
LocationCliquez ou appuyez ici pour entrer du texte.

**Type of intensive care unit activity**

Medical ☐ Surgical ☐ Polyvalent ☐

2. **Date and time of completion of the questionnaire**

| Date/Time | Passage order | Questionnaire Code |
|---|---|---|
| ☐ | ☐ | ☐ |

3a. **Profession**

☐Physician (senior) ☐Physician (internal) ☐Nurses ☐Auxiliary nurses ☐ Physical Therapist

**3b. <u>Are-you allocated to</u>**

☐Intensive care unit ☐Continuous care unit ☐both

**4. <u>Gender</u>**

☐Male ☐Female

**5. <u>Age and experience</u>**

Age ☐

Experience in profession ☐

Experience in current position ☐

**6. <u>Working quota</u>**

In full-time % ☐

**7a. Over <u>the past 30 days, what was your primary allocation?</u>**

☐Day ☐Afternoon (if 3-8) ☐Night

**7b. Over <u>the past 30 days, you were allocated to this schedule</u>**

☐All the time ☐Mostly

**1. <u>Recent Work history</u>**

Start time of current shift ☐

**Were you at work during the last 3 days?**

| D-3 | D-2 | D-1 |
|---|---|---|
| ☐Yes ☐No | ☐Yes ☐No | ☐Yes ☐No |

**2. <u>Breaks usually taken over a worked shift</u>**

Number of breaks ☐ average duration (in min) ☐

**3. <u>Over the last 3 days worked, did you skip a break due to workload?</u>**

☐Yes ☐No

**4. <u>Marital status</u>**

☐Single ☐ Married or partnered ☐ Separated-widowed-divorced

**5. <u>Do you have children?</u>**

☐Yes ☐No

**if yes, number of children:** Less than 2 years old ☐
From 2 to 10 years old ☐
More than 10 years old ☐

**6. <u>Commuting duration</u>**

Total time (in min) ☐

7. <u>Nottingham Health Profile:</u> **For each of the following statements, indicate whether they describe your current state (close to life in general)**

**I'm tired all the time**
☐Yes ☐No

**I take pills to help me sleep**
☐Yes ☐Non

**Everything is an effort**
☐Yes ☐No

**I wake up very early and have trouble sleeping**
☐Yes ☐No

**I lie awake for most of the night**
☐Yes ☐No

**I get tired very quickly**
☐Yes ☐No

**It takes me a long time to get to sleep**
☐Yes ☐No

**I sleep badly at night**
☐Yes ☐No

8. **<u>How are you feeling right now?</u>**
☐Exhausted ☐Tired ☐Somewhat healthy ☐In excellent condition

9. **<u>Did you wake up today feeling tired?</u>**
☐Yes ☐No

10. **<u>Sleep history over the last 24 hours</u>**

| | |
|---|---|
| Time at which you woke up this day (excluding nap) | |
| Bedtime | |
| Total duration of naps (hr :min) | |

11. **<u>How often do you…</u>**
**Work different hours than originally planned**
☐Never ☐Occasionally ☐Often ☐Very often

**Work overtime**
☐Never ☐Occasionally ☐Often ☐Very often

12. **<u>PSS-10</u> : In the last month, how often have you …**

**been upset because of something that happened unexpectedly?**
☐Never ☐Almost never ☐Sometimes ☐Fairly often ☐Very often

**Felt that you were unable to control the important things in your life?**
☐Never ☐Almost never ☐Sometimes ☐Fairly often ☐Very often

**Felt nervous and “stressed”?**
☐Never ☐Almost never ☐Sometimes ☐Fairly often ☐Very often

**Felt confident about your ability to handle your personal problems?**
☐Never ☐Almost never ☐Sometimes ☐Fairly often ☐Very often

**felt that things were going your way?**
☐Never ☐Almost never ☐Sometimes ☐Fairly often ☐Very often

**found that you could not cope with all the things that you had to do?**
☐Never ☐Almost never ☐Sometimes ☐Fairly often ☐Very often

**been able to control irritations in your life?**
☐Never ☐Almost never ☐Sometimes ☐Fairly often ☐Very often

**Felt that you were on top of things?**
☐Never ☐Almost never ☐Sometimes ☐Fairly often ☐Very often

**Been angered because of things that were outside of your control?**
☐Never ☐Almost never ☐Sometimes ☐Fairly often ☐Very often

**Felt difficulties were piling up so high that you could not overcome them?**
☐Never ☐Almost never ☐Sometimes ☐Fairly often ☐Very often

13. **Questionnaire de Karasek : your opinion on your working situation**
**My superior feels concerned by the well-being of his subordinates**
☐Fully disagree ☐Disagree ☐Agree ☐Fully agree

**My superior pays attention to what I am saying**
☐Fully disagree ☐Disagree ☐Agree ☐Fully agree

**My superior helps me carry out my tasks**
☐Fully disagree ☐Disagree ☐Agree ☐Fully agree

**My superior manages to make his subordinates collaborate**
☐Fully disagree ☐Disagree ☐Agree ☐Fully agree

**My coworkers are professionally competent**
☐Fully disagree ☐Disagree ☐Agree ☐Fully agree

**My coworkers are interested in me**
☐Fully disagree ☐Disagree ☐Agree ☐Fully agree

**My coworkers are friendly**
☐Fully disagree ☐Disagree ☐Agree ☐Fully agree

**My coworkers help me carry out my tasks**
☐Fully disagree ☐Disagree ☐Agree ☐Fully agree
